# Curating the Fetal Genome: Experience of the ClinGen Prenatal Gene Curation Expert Panel (GCEP)

**DOI:** 10.1002/pd.70246

**Published:** 2026-09-23

**Authors:** Stephanie N. Galloway, Jessica L. Giordano, Carrie Shawber, Natalie Chandler, Lauren Westerfield, Prenatal GCEP Panel members, Ronald J. Wapner, Wendy Chung, Ignatia B. Van den Veyver, Shagun Aggarwal, Caitlin Baptiste, Brenna Boyd, Amanda Buchanan, Alicia Byrne, Imen Chakchouk, David Chitayat, Lyn Chitty, Pinal Dalwadi, Avinash Dharmadhikari, Lilian Downie, Stephanie Dukhovny, Mythily Ganapathi, Kelly Gilmore, Elena Greenfeld, Lylach Haizler‐Cohen, Vaidehi Jobanputra, Gabrielle Litteken, Hannah Perrin, Julie Ratliff, Rebecca Reimers, Erin Riggs, Teresa Sparks, Samantha Stover, Amanda Thomas‐Wilson, Leandra Tolusso, Neeta Vora, Tsegaselassie Workalemahu, Roni Zemet‐Lazar

**Affiliations:** ^1^ Department of Obstetrics and Gynecology Columbia University Irving Medical Center New York New York USA; ^2^ North Thames NHS Regional Genetics Service Great Ormond Street NHS Foundation Trust London UK; ^3^ Department of Obstetrics & Gynecology Baylor College of Medicine Houston Texas USA; ^4^ Department of Molecular & Human Genetics Baylor College of Medicine Houston Texas USA; ^5^ Texas Children's Hospital Houston Texas USA; ^6^ A complete list of members and affiliations appears in Supporting Information S1; ^7^ Department of Pediatrics Columbia University Irving Medical Center New York New York USA; ^8^ Department of Medicine Columbia University Irving Medical Center New York New York USA; ^9^ Department of Pediatrics Boston Children's Hospital Boston Massachusetts USA; ^10^ Harvard Medical School Boston Massachusetts USA

## Abstract

**Objective:**

Expert prenatal focused gene‐disease curation is necessary to accurately inform clinical care in the setting of rapidly expanding prenatal genomic sequencing.

**Methods:**

An international Prenatal Gene Curation Expert Panel assembled and systematically reviewed genes asserted to be associated with prenatal hydrops, stillbirth, or severe anomalies using the ClinGen framework.

**Results:**

The ClinGen Prenatal GCEP published evidence summaries for 63 disease relationships involving 61 genes with unique and clinically severe prenatally observed fetal phenotypes.

**Conclusion:**

The ClinGen Prenatal GCEP work addresses a gap in genomic medicine by deepening our understanding of lethal and severe prenatal phenotypes, while supporting accurate diagnosis, counseling, and future treatment strategies in prenatal care.

## Key Summary

- What's already known about this topic?
  ◦ Knowledge about gene‐disease relationships is less robust prenatally due to limitations of fetal phenotype descriptions and knowledge about genomic data associated with prenatal phenotypes.
  ◦ This makes interpreting the causality of identified genes and variants more challenging for prenatal genomic testing compared with testing after birth in children and adults.
- What does this study add?
  ◦ We report the first experience with curation of prenatal gene‐disease relationships using the standardized Clinical Genome Resource (ClinGen) evidence framework.
  ◦ Integrating specialized prenatal expert review within gene‐disease relationship curations can fill the current knowledge gaps in this area. This will be essential for advancing prenatal precision genomic medicine.

## 1 Introduction

Fetal structural anomalies and unique fetal phenotypes such as non‐immune hydrops fetalis (NIHF) and stillbirth, affecting between 1% and 3% of all pregnancies, are leading causes of severe disability, fetal demise, and postnatal death. Major fetal structural anomalies complicate 2%–3% of all pregnancies, and up to 40% have an identifiable genetic cause. Increasing resolution and precision of diagnostic imaging by prenatal ultrasound and fetal magnetic resonance imaging (MRI), paired with innovations in prenatal genetic testing, such as chromosomal microarray analysis, gene panels, exome, and genome sequencing, improve our ability to identify the cause of fetal anomalies [1, 2]. Among those, NIHF presents a significant diagnostic challenge. Recent sequencing studies have identified a genetic cause for more than 29% of fetal hydrops of unknown origin, of which many were variants in genes not previously known to be associated with this severe fetal phenotype [3]. Stillbirth is one of the most common adverse outcomes of pregnancy, with approximately 3.04 million stillbirths occurring worldwide in 2021 [4]. Stanley et al. found that 8%–9% of unexplained stillbirths had pathogenic or likely pathogenic variants in genes known to cause neonatal, childhood, or adult disorders, and that another 6%–7% have variants in genes critical for *in utero* survival, but not currently associated with postnatal disease [5].

The full benefit of prenatal genome sequencing is, however, still not realized due to the difficulty in interpreting the pathogenicity of identified variants in the presence of incomplete fetal phenotype information. This limits the accuracy of prenatal counseling about prognosis, recurrence risk, and reproductive options, as well as planning pre‐, peri‐, and neonatal clinical management. To address this issue, it is critical to systematically evaluate how genetic variants identified through prenatal sequencing relate to the fetal phenotype, but this process presents unique challenges. Prenatal phenotyping is hampered by the dearth of functional studies and the limitations of assessing dysmorphology by prenatal imaging. Observed fetal phenotypes of known genetic conditions can also differ from the condition's presentation in children or adults. Prenatal phenotypes can evolve and even disappear as gestation progresses or only present prenatally because the condition is lethal and therefore never observed in liveborn infants. Experience with prenatal genetic sequencing for stillbirths is also limited. Thus, current literature and genetic databases that are queried to assist with the interpretation of genetic variants mainly include data from childhood and adult genotype‐phenotype correlations. However, the correct interpretation of genomic variants with prenatal presentations of genetic disease is crucial for expanding our knowledge of the full phenotypic spectrum of genetic disorders and consequently for accurate prenatal diagnosis.

Different community collaborative efforts to understand and assess the evidence for gene‐disease relationships have been developed in response to the growing list of genetic diseases. These include PanelApp [6], with both the United Kingdom (UK) and Australian versions [7], the Gene Curation Coalition (GenCC) [8], and the Clinical Genome Resource (ClinGen) [9], but all were initially highly focused on postnatal genotype‐phenotype correlation. The Clinical Genome Resource (ClinGen) is a United States National Institutes of Health (NIH)–funded consortium dedicated to clarifying the clinical relevance of genes and genetic variants for use in patient diagnosis, research, and precision medicine [9]. Its goals are to: “aggregate clinical and genetic data; curate evidence according to standardized frameworks; disseminate expert reviewed knowledge; and continuously evaluate and improve our work to achieve the greatest impact.” [10].

Here, we report how we addressed the need for dedicated curation of the evidence for prenatal genotype‐phenotype relationships by assembling a new ClinGen Prenatal Gene Curation Expert Panel (GCEP). This Prenatal GCEP currently focuses on evaluating genes implicated in uniquely prenatal phenotypes of non‐immune hydrops fetalis, unexplained stillbirth, and other genetic conditions with distinct prenatal presentations. The Prenatal GCEP is among > 45 other disease or phenotype specific GCEPs. We describe here our standard processes, progress, challenges, and lessons learned since its implementation in 2021. We describe its place among other international efforts, including the PanelApp Fetal Anomalies Panel from Genomics England and the Fetal Genotype:Phenotype Reports (FGPR) published in the new curation corner in *Prenatal Diagnosis*.

## 2 Methods

### 2.1 Selection of the Curation Gene List

The Prenatal GCEP focuses on genes associated with phenotypes unique to pregnancy, such as non‐immune hydrops fetalis (NIHF), stillbirth, and distinct prenatal structural anomalies. The initial NIHF gene list for curation was assembled in 2020 and contains 151 genes from a review of three commercially available and frequently used NIHF diagnostic gene panels (with 52–130 genes), the PanelApp UK Fetal Anomalies Panel (89 genes) [6], and genes diagnosed through the NIHF repository at the University of California, San Francisco (provided by an expert panel member). The initial stillbirth curation gene list contained 221 genes based on an exhaustive search of the OMIM database [11] for stillbirth‐related genes, Columbia University Irving Medical Center's experience with sequencing 246 stillbirth cases, review of stillbirths from the Fetal Anomalies Panel of the PAGE consortium [2], and 94 cardiac‐related sudden death‐susceptibility genes reported in a series of stillbirths [5, 12]. Additional genes were added over time with new discoveries through clinical care or publications, or recommendations by other GCEPs who encountered prenatal phenotypes during their curation work.

### 2.2 Composition of the Prenatal GCEP

The implementation of this GCEP and training of expert members and biocurators was conducted and maintained according to ClinGen's detailed protocol [10, 13]. Prenatal GCEP members and biocurators are a multinational group of early career, midcareer, and senior faculty experts in clinical genetics, maternal‐fetal medicine, pediatrics, and molecular genetic diagnostics. Current members represent 23 institutions from 5 countries (United States, Canada, England, Australia, and India). All new biocurators attend four meetings before they are assigned their first curation, which they conduct with oversight of an expert and support of an experienced ClinGen biocurator. The Prenatal GCEP meets weekly at alternating times to accommodate time zones of participants from different continents.

### 2.3 Gene Curation Process

We conduct all curations using previously published data according to the standard operations procedures for the rigorous ClinGen gene‐disease validity curation framework described by Strande et al., which combines qualitative and semiquantitative measures to score available genetic and experimental evidence for gene‐disease relationships to arrive at a classification [13]. We assign a single gene to a biocurator and clinical expert pair at least 4 weeks before the meeting when they are scheduled to present. They complete a thorough literature review and systematic evidence evaluation using the ClinGen framework, which they then present at the weekly meeting for feedback from the other Prenatal GCEP experts and biocurators.

We follow two approaches for gene curation (Figure 1): *primary full curations* for genes not previously formally curated by another ClinGen GCEP and *secondary curations* which contribute missing or new prenatal information to genes that were previously curated by other GCEPs for phenotypes at other stages of life. The first step of a full curation is the *pre‐curation* to determine if multiple asserted diseases caused by variants in a gene are indeed distinct disease entities that should be separately curated (*splitting*), or alternatively, whether different asserted diseases represent a single phenotype or disease relationship and should be curated together under a common disease name (*lumping*). *Splitting* is favored for clearly distinguishable phenotypes that typically result from different modes of action of variants (e.g., dominant gain of function vs. recessive loss of function) [14]. *Lumping* is favored when phenotypes associated with a gene represent a spectrum or continuum of the same condition that typically is caused by similar types of variants. The pre‐curation and lumping versus splitting decisions are documented in the ClinGen Gene Tracker as well as in the final gene curation summary.

**FIGURE 1:**
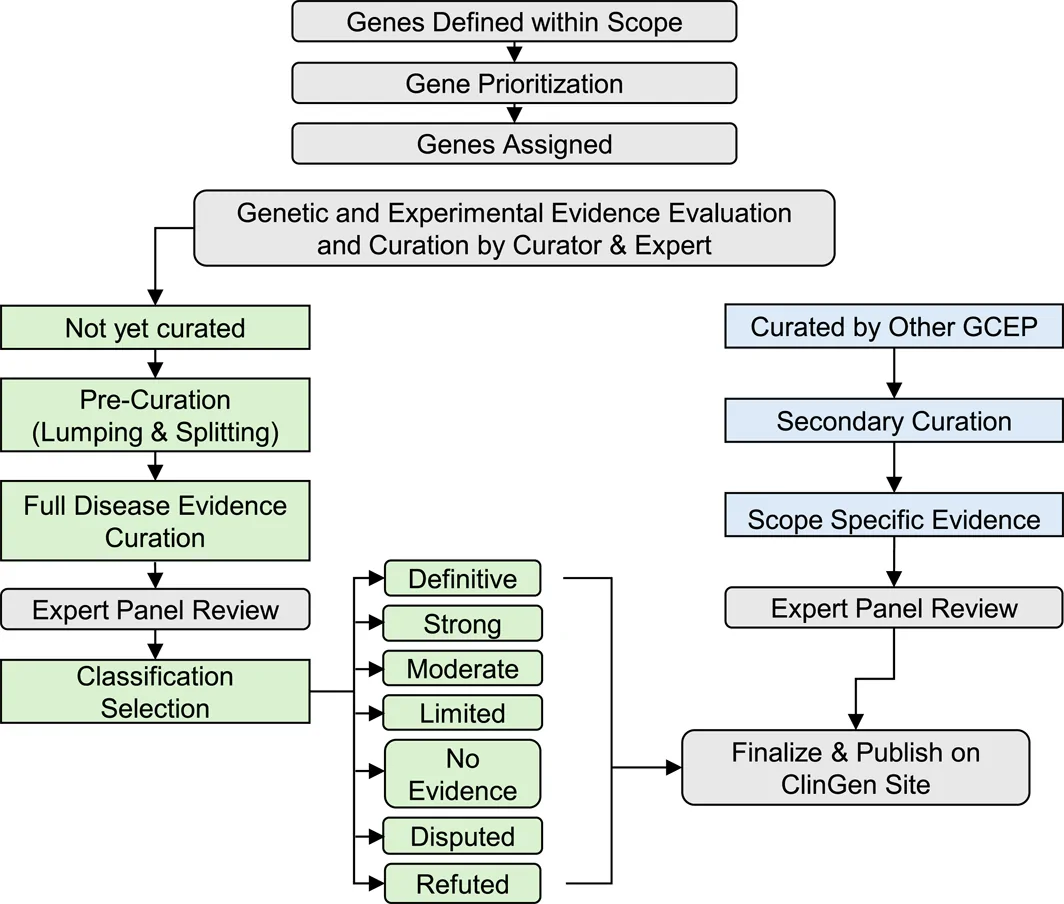
Gene curation process. This figure illustrates the two approaches used for gene curation. Primary curations are performed for genes not previously curated by another gene curation expert panel (GCEP), beginning with pre‐curation to determine whether asserted diseases should be lumped or split, followed by full curation and expert panel review to assign a final disease classification. Secondary curations are conducted for genes previously curated by other GCEPs with a focus on prenatal phenotypes. Findings are communicated back to the primary GCEP for integration into their curation.

After the pre‐curation is complete, a full curation is undertaken [10, 13]. For this we use the ClinGen framework to evaluate variants in the gene of interest and any associated experimental data reported for patients with the disease in published case reports, family studies, case‐control studies, and publications with experimental data, such as biochemical assays, gene and protein expression data, or other functional studies in cell‐based and animal disease models. The systematic scoring algorithm can yield a maximum of 18 points, derived from 12 points for genetic evidence and 6 points for experimental evidence. Gene‐disease relationships are then classified based on the strength of supportive evidence as follows: definitive (12–18 points, with replication over time, defined as a minimum of three years), strong (12–18 points), moderate (7–11 points), limited (0.1–6 points), no known disease relationship (no available reports that directly implicate the gene in human disease cases), disputed (conflicting evidence for a gene‐disease relationship), and refuted (no valid evidence for a gene disease relationship). The biocurator presents precurations and curations using slides to the assembled panel during a virtual meeting. Panel quorum is achieved when at least 9 members are in attendance. For step 1 of the full curation (genetic evidence), the biocurator presents the clinical and genetic data for each scored variant and the preliminary score they assigned. The panel members comment in real‐time on whether they agree or disagree with the assigned score. If there is disagreement, consensus is reached during the meeting and the score is updated as needed. This is repeated for each presented scored variant until maximum score for genetic evidence is reached. The same process is followed for step 2, functional evidence scoring, and for the final score and classification. The panel members then review the evidence summary presented by the biocurator which is edited in real time. If for any of these more research or further review is needed, follow‐up communication and approval of any changes is done by e‐mail. Rarely, the curation is re‐presented at a future meeting taking into account discussions and recommendations from the panel. After final agreement of the scoring by the GCEP experts and panel members, scores are recorded in ClinGen's gene curation interface and a summary is published on the Prenatal GCEP page of the ClinGen website [15]. Curation status for individual genes can also be searched from the ClinGen landing page.

The secondary curation approach recognizes that uniquely prenatal phenotypes, like NIHF or stillbirth, can be not‐yet‐recognized features of a syndromic condition. If the causative gene for that syndrome was previously formally curated by another GCEP, the Prenatal GCEP will perform a secondary curation focused on the prenatal phenotype. We apply the same rigor in reviewing genetic and experimental evidence for these phenotypes, but without a full repeat scoring. Instead, a secondary curation summary is sent to the primary GCEP, who will incorporate it into their curation summary. The prenatal GCEP chairs and coordinators communicate with these GCEPs for questions and input. After approval by their panel, the primary GCEP who will post our secondary curation summary as an addendum to their summary. Disagreements or needs for shared expertise are resolved through dedicated meetings with representatives of other panels included. For not yet curated syndromic genes listed as “of interest” or “in scope” by another GCEP or genes nominated for curation by others who contact ClinGen or our coordinators, the prenatal GCEP coordinators communicate with that GCEP for agreement on whether we will conduct the primary curation or contribute prenatal phenotype aspects to their curation.

### 2.4 Strategies for Gene Prioritization

Because the original gene list for our Prenatal GCEP is extensive and new genes related to unique prenatal phenotypes are identified and reported on an ongoing basis, we devised two main strategies to prioritize gene assignment for curator‐expert pairs. For the first strategy, GCEP coordinators assign five genes from our existing gene list and any new genes nominated for curation by panel experts or others to the co‐chairs and selected GCEP members for an initial review to rank them in order of priority and decide on primary full versus secondary curation needs. Priority ranking considers prior curation efforts, reports of confirmed cardinal prenatal phenotypes, and known inclusion of the gene on panels clinically available for prenatal diagnosis or screening. We also review if the gene is included on the English nationally sanctioned NHS Genomic Medicine Service (GMS) “Fetal anomalies with a likely genetic cause” panel [16]. Finally, we cross‐reference listed genes with genes published in the Fetal Genotype:Phenotype Reports (FGPR) in *Prenatal Diagnosis*. These proposed rankings are reviewed for consensus during a dedicated meeting. We also follow the ClinGen guidelines for scheduling recuration of genes for which the evidence is less than definitive [13].

## 3 Results

### 3.1 Current Experience With Prenatal GCEP Gene Curation

During the first year, while the GCEP members became accustomed to the curation principles and more discussion and input from ClinGen experts was required, we scheduled one gene curation per meeting. Occasionally, a follow‐up meeting was required to resolve questions. We have since transitioned to two curations per meeting to increase the number of curated genes (Figure 2). At the time of this report, we have 63 gene‐disease relationships published on the ClinGen website (Table S1) involving 61 genes (*FLT4* and *PIEZO1* were curated for two diseases). Of these, 31 were primary full curations resulting in 29 definitive and 2 moderate classifications and 32 were secondary curations (Table 1).

**FIGURE 2:**
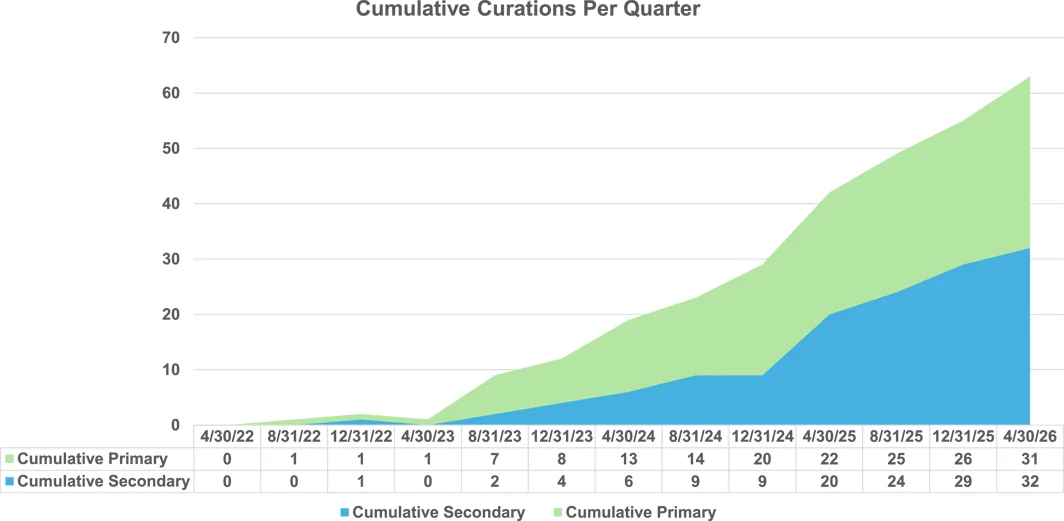
Cumulative progress of the prenatal gene curation expert panel. This graph illustrates the cumulative number of curations completed each quarter across two curation categories (primary curation and secondary curations) and publications on the ClinGen website. The data points on the graph reflect quarterly accrued cumulative counts within each category. The accompanying table at the bottom displays the absolute cumulative values. Early in the panel's efforts, curations progressed at a slower pace as the team worked through one gene per meeting to gain familiarity with the ClinGen framework. As experience increased, the team established a sustainable workflow, resulting in a steady increase in curations and meaningful contributions to the existing genomic knowledge base.

**TABLE 1:** Total number of gene‐disease curations performed using associated evidence.

Curation	Result	Evidence	*N* (%)
Full	Definitive	12–18 points, with replication over time (≥ 3 years)	29 (46%)
Full	Strong	12–18 points	0
Full	Moderate	7‐11 points	2 (3%)
Full	Limited	0.1–6 points	0
Full	No relationship	No available reports implicating gene in the curated disease	0
Full	Disputed	Conflicting evidence for gene‐disease relationship	0
Full	Refuted	No valid evidence for gene disease relationship	0
Secondary	Per prior curation	Prenatal features added	32 (51%)
Total			63

*Note:* We curated 61 genes for 63 prenatal phenotypes. Two genes (*FLT4* and *PIEZO1*) were curated for 2 diseases with prenatal phenotypes.

Of the 41 genes curated because of asserted causality for hydrops, 17 were also previously linked to stillbirth. The gene‐disease relationships for 31 of these 41 genes included hydrops as a phenotype, but for 9 of the 41 (*ACE, CANT1, COG6, DHCR7, GNPTAB, MVK, MGAT2, PHGDH, TRIP11*; see supplemental table 1) we could not confirm from reviewing the evidence that the significant prenatal phenotypic features included hydrops. Additionally, we provided a secondary curation for four of these 41 genes (*WDFY3, MGAT2, SMPD1, FIG4*) but found no evidence of specific variants associated with prenatal phenotypes. Our detailed curation efforts found no evidence for any prenatal phenotypes associated with *WDFY3* and revealed that the publications about *FIG4*, *MGAT2, and SMPD1* that reported prenatal phenotypes included cases with only a clinical diagnosis without molecular confirmation, preventing scoring of the molecular evidence of the gene‐disease relationship.

Of the 37 genes curated because of an asserted causal relationship to stillbirth, 20 had sufficient supporting evidence for stillbirth. For the remaining 17 genes, we found no evidence or molecular confirmation for a relationship with this phenotype. An example is our secondary curation for X‐linked *TAZ (TAFAZZIN)*, the gene associated with Barth syndrome (OMIM #302060), characterized by cardiomyopathy, neutropenia, skeletal myopathy, and prepubertal growth delay in males. Although we found several reports of higher prevalence of stillbirths in carrier families, there were no molecular testing data for most losses. Even if clinical evidence is strong, obtaining molecular testing on fetal losses in families with histories of stillbirth is often not possible, which prevented us from reaching a firm conclusion that there is an increased risk of miscarriage or demise in affected male pregnancies.

### 3.2 Comparison to the PanelApp UK Fetal Anomaly Panel and Fetal Genotype‐Phenotype Reports

The genes included on the PanelApp UK “Fetal anomalies with a likely genetic cause” panel are reviewed by a panel of experts for evidence of a prenatal phenotype and meeting eligibility criteria for testing according to the NHS Genomic Medicine Service test directory [17]. The genes are scored by a traffic light system to reflect the strength of evidence for disease association under a specified mode of inheritance—Green: high evidence of a gene presenting with a relevant phenotype, for clinical use; Amber: moderate evidence, not for clinical use; Red: low evidence, not for clinical use. To be rated as a “*Green*” gene, pathogenic variants must have been seen in three or more unrelated families or in two to three unrelated families with strong additional functional data. We identified 247 genes in our current curation list that overlap with the PanelApp Fetal Anomalies panel version 6.185 [16]. Of those overlapping genes, we have published 55, with more in progress. The Prenatal GCEP's curation conclusions are in concordance with the PanelApp classifications, except for the gene *HYLS1,* associated with hydrolethalus syndrome (OMIM 236680). The ClinGen classification yielded a moderate gene‐disease relationship because most reported cases have a recurrent variant, limiting the ability to score the genetic evidence within the current ClinGen framework. While more evidence is needed to definitively establish this relationship, there is no convincing contradictory evidence in the literature.

We also cross‐reference our curation gene list with genes published in the Fetal Genotype: Phenotype Reports (FGPR) in *Prenatal Diagnosis*. At the time of preparation of this report, there have been 79 FGPRs published on 76 genes with new or complex prenatal phenotype‐genotype relationships. Among those, 9 have been published on the ClinGen website by the prenatal GCEP (five primary and four secondary curations), and 14 are listed to be curated in the future. All the primary curations reached definitive classifications, and the secondary curations confirmed medical evidence of prenatal phenotypes. While none of the gene‐disease relationships were novel, the FGPR publications assisted our curations by clarifying or supporting the scope of prenatal presentations.

## 4 Discussion

Through a multidisciplinary effort, the Prenatal Gene Curation Expert Panel has published 60 gene‐disease relationships associated with unique and clinically severe prenatally observed fetal phenotypes. As shown in Figure 2, productivity continues to increase as our experience grows. This effort is timely given that genomic sequencing is increasingly used for prenatal diagnosis. Although ClinGen, first launched in 2013, has curated over 3300 unique genes through the work of multiple GCEPs, there was no prenatally focused GCEP before October 2021. This led to underrepresentation of curations of genes associated with uniquely prenatal phenotypes. In addition, curations conducted by disease or disease‐class‐specific GCEPs do not always describe the prenatal presentations that could represent the severe end of the phenotypic spectrum of these conditions. Addressing this limitation motivated us to also focus on secondary curations to include missing prenatal features in the summaries of already curated genes by other GCEPs. A representative example is the curation of *CTSA*. Its primary curation was expertly completed by the Lysosomal GCEP who reviewed postnatal cases to investigate the gene‐disease relationship. Our prenatal secondary curation added evidence of the “early infantile” presentation which is associated with pre‐ or perinatal lethality. We also learned along the way that prenatal curation comes with unique challenges which highlights the value of this effort, but also the importance of integration with other ongoing initiatives to improve the classification of disease genes and variants associated with prenatally detectable fetal phenotypes.

### 4.1 Challenges With Prenatal Gene Curation

The available information on prenatal phenotypes of genetic disorders is still incomplete, making it more challenging to reach highly scored genetic evidence. An important reason for this is the limited or absent documentation of prenatal fetal and placental features in published case reports or series of newly diagnosed genetic syndromes in children. This could be because it is challenging to collect this information retrospectively when a diagnosis is made in a child, along with less precise prenatal imaging when a diagnosis is not suspected prenatally. Some genetic conditions are prenatally lethal, and when stillbirth occurs, samples for genetic analysis are not always collected, precluding diagnosis and consequently underreporting of prenatally lethal genetic disorders. For other genetic conditions, the prenatal phenotype or pregnancy loss may be a previously unrecognized or unappreciated severe presentation of the disease, making it difficult to confirm the molecular diagnosis and association.

Although prenatal genetic testing has evolved significantly in recent years from strategies such as karyotyping to high‐throughput sequencing methods, genomic sequencing tests are not yet widely used, in part due to cost and variable access and guidelines across the world. As a result, many genetic disorders are not prenatally diagnosed, which limits the knowledge about prenatal phenotypes of genetic disorders that is required for effective prenatal gene curation. We faced this with the secondary curation of *ASAH1,* a gene with a primary curation with a “definitive” classification for its association with sphingolipidosis. While this condition can present prenatally with contractures and hydrops, our secondary curation found only a small number of reports of prenatal cases, of which few had genetic testing [18, 19].

Biochemical testing and other functional assays, which can be the cornerstone for postnatal diagnosis of genetic conditions with a metabolic phenotype are very limited prenatally. Conversely, for some genetic conditions there may be reports with only biochemical test results that predated the discovery of a causative gene and potential expansion of the associated phenotype to include features observable by prenatal ultrasound. We experienced this difficulty with the secondary curation of *SMPD1* for its association with NIHF. There were just two reports of prenatal *SMPD1*‐related acid sphingomyelinase deficiency (Niemann Pick A/B) presenting as NIHF and biochemical testing suggestive of Niemann Pick, but without molecular confirmation [20, 21]. This could indicate that this condition is underdiagnosed prenatally, or alternatively, that it rarely presents prenatally, but this distinction cannot currently be resolved.

These are representative examples of curations we aim to re‐review in the future, according to the ClinGen recuration schedule or when significantly more information becomes available.

### 4.2 Strategies to Address Prenatal Gene Curation Challenges

We anticipated some of these described challenges, and they motivated our initial focus on well‐described clinical prenatal phenotypes with genetic etiologies, NIHF and stillbirth. We further prioritized hydrops‐associated genes present on several hydrops diagnostic gene panels. To date, all our completed full curations for genes linked to hydrops are on two or more of these hydrops panels.

To address limited descriptions of prenatal phenotypes of already curated genes, we created prenatally focused secondary curations to expand ClinGen's resources across the entire lifespan. This led ClinGen leaders to recognize the value of prenatal phenotypes in many genetic syndromes being curated and led them to expand the utilization of the secondary curation concept. As a result, we currently collaborate with many other GCEPS, such as the Skeletal Disorders GCEP, the General Inborn Errors of Metabolism GCEP, and the Intellectual Disability and Autism GCEP. In turn, we incorporate other secondary analysis from other specialties into our primary prenatal GCEP curations.

Another challenge, not unique to prenatal gene‐disease validity curation, is that while expert‐panel driven curation is thorough with different levels of review, increasing the number of curations is desirable. To this effect, the ClinGen consortium is exploring scalability through automation. If or when the consortium develops or recommends solutions for scalability, we plan to adopt them.

### 4.3 Synergy With Other Efforts to Validate Prenatal Genotype‐Phenotype Associations

To maximize the available information about the genes we prioritized for prenatal curation, we compared the prenatal GCEP's list of genes with the UK PanelApp fetal anomalies panel, which is reviewed twice annually by expert reviewers who conduct literature searches, check for novel gene‐phenotype relationships in OMIM, and verify new entries from other relevant panels within PanelApp. However, this review does not use a formal scoring system framework such as ClinGen for gene curation. An average of 94 genes were added in each of the last three reviews of the PanelApp fetal anomalies panel, which underscores the challenges of keeping up with novel gene‐disease association reports in the literature. A key difference in the PanelApp reviews to our prenatal ClinGen review is that the PanelApp evidence does not require being specifically reported prenatally, but genes are added if associated phenotypic features seen postnatally might have been present prenatally (e.g., agenesis of the corpus collosum). For phenotypes such as microcephaly, which can have a prenatal or postnatal onset, it can be difficult to determine if the phenotype could have been observed prenatally, particularly because most publications of cohorts of patients with a newly discovered genetic disease usually describe phenotypes from the time of birth and rarely include a review of prenatal findings.

The prenatal GCEP advocates for the continued expansion of publications of prenatal genomic findings as they contain the data that form the basis for gene curation using the ClinGen framework. We encourage members to submit cases for publication, for example, as Fetal Genotype‐Phenotype Reports. We also encourage collaboration on publications of case series [22].

Now that more prenatal sequencing is being done for pregnancies with fetal anomalies and more recently, also for pregnancies without fetal phenotypes, we encourage the global clinical and laboratory prenatal genetic diagnosis community to accelerate contributions to the knowledge base on prenatal genotype‐phenotype correlations by reporting on new and interesting findings using the different pathways available. These include depositing sequence variants and associated clinical prenatal data into ClinVar, which is the primary resource for aggregated information about the relationship of genomic variation to human disease [23]. Another growing opportunity to add to the knowledgebase includes the deposition of sequence and clinical data in the cloud‐based repository of the International Fetal Genomics Consortium (IFGC) [24].

The knowledge base on prenatal phenotypes and genes relevant to stillbirth is also growing thanks to research efforts focused on cataloging the human intolerome genes relevant to recurrent pregnancy loss and genes relevant to lethal phenotypes [25, 26, 27]. We hope and expect that future clinical prenatal sequencing interpretations and published reports of sequenced pregnancies will consider such important data sources, so that they can lead to new diagnoses reported in the literature, for example in FGPR and in ClinVar. In turn, these will be accessible as evidence for new gene‐disease validity curations by our GCEP that is usable according to the ClinGen gene‐disease validity standard operating procedures [13]. Finally, clinicians can also recommend that patients themselves take an active role in their data‐sharing by enrolling in ClinGen's patient repository, GenomeConnect (genomeconnect.org). Patients can consent online and provide information on relevant fetal phenotypes as well as longitudinal follow‐up information. With consent, their reported variants are shared with ClinVar, and the sequence files can also be shared with the IFGC repository, making them available for periodic reanalysis on a research basis [28, 29, 30].

## 5 Conclusion

The work of the ClinGen Prenatal Gene Curation Expert Panel highlights the critical importance of rigorous, prenatal‐focused gene–disease curation in an era of rapidly expanding use of prenatal genomic sequencing in clinical care. By systematically evaluating genes implicated in NIHF and stillbirth, the Prenatal GCEP has addressed a major gap in genomic medicine: the limited and often fragmented understanding of how genetic disorders manifest in the fetus. Since its inception, the Prenatal GCEP, working within ClinGen's robust collaborative framework, has generated a substantial body of curated gene‐disease relationships and integrated prenatal‐specific phenotypic insights into the broader ClinGen ecosystem, thereby directly supporting more accurate interpretation of prenatal genetic testing and informed clinical decision‐making. Ultimately, these efforts will deepen understanding of lethal and severe prenatal phenotypes, improve diagnostic accuracy and clinical utility of prenatal genome sequencing, together laying the groundwork for precision counseling, targeted management, and future therapeutic strategies in prenatal care.

## Funding

This publication was supported in part by the National Human Genome Research Institute (grant U24HG006834) and the Eunice Kennedy Shriver National Institute of Child Health and Human Development (grant U24HD104588) of the National Institutes of Health. The content is solely the responsibility of the authors and does not necessarily represent the official views of the National Institutes of Health.

## Ethics Statement

The authors have nothing to report.

## Consent

The authors have nothing to report.

## Conflicts of Interest

Dr. Van den Veyver is an associate editor and Dr. Chandler is a junior editor of *Prenatal Diagnosis*.

## Supporting information


Supporting Information S1

**Table S1:** Gene‐disease curations by the Prenatal GCEP.

## Data Availability

The data that support the findings of this study are openly available in the Clinical Genome Resource at https://www.clinicalgenome.org/affiliation/40106/.
