# Supplementary material for: Curating the Fetal Genome: Experience of the ClinGen Prenatal Gene Curation Expert Panel (GCEP)
Source: Prenat Diagn. 2026 Sep 23;46(11):1703–10. doi: 10.1002/pd.70246 (PMC13659245; doi:10.1002/pd.70246)
Supplement: Supplementary file 1 [file PD-46-1703-s001.docx]

**Supplemental Table 1**: **Gene-Disease curations by the Prenatal GCEP.**

The table lists all full curations (N=31) with strength of evidence scores (definitive N=29; moderate N=2) and all secondary curations (N=32) for gene-disease relationships with the evaluated relevant prenatal phenotypes. Included are curations published to the ClinGen website as of submission of this article. The asserted phenotypes, Non-Immune Hydrops Fetalis (NIHF) and Stillbirth) are in respective columns. The + or – in “Confirmed association” columns indicates whether literature review and curation found evidence (+) or not (-) for the asserted phenotype. In the “Confirmed Anomalies” column + or - indicates that there were prenatally detectable fetal anomalies that were confirmed during curation. & indicates that there were clinically described or biochemical data available, but without linked molecular evidence, or that only very cases were reported. We note that for two genes, *PIEZO1* and *FLT4*, we curated two associated diseases.

| Gene | Name of Curated Disease | NIHF | Confirmed association | Stillbirth | Confirmed association | Confirmed anomalies | Curation Type: Classification |
| --- | --- | --- | --- | --- | --- | --- | --- |
| *ACE* | Renal tubular dysgenesis | + | - | + | + | + | Full: Definitive |
| *AMER1* | Osteopathia striata with cranial sclerosis | - | - | + | + | + | Full: Definitive |
| *ARSL* | Chondrodysplasia punctata, X-linked recessive | - | - | + | - | + | Secondary |
| *ASAH1* | Farber lipogranulomatosis; Spinal muscular atrophy with progressive myoclonic epilepsy | + | + | - | - | + | Secondary |
| *ATP1A2* | Fetal akinesia, respiratory insufficiency, microcephaly, polymicrogyria, and dysmorphic facies | + | + | - | - | + | Full: Definitive |
| *CANT1* | Epiphyseal dysplasia, multiple, 7; Desbuquois dysplasia 1 | + | - | - | - | + | Full: Definitive |
| *CDAN1* | Dyserythropoietic anemia, congenital, type Ia | + | + | - | - | + | Full: Definitive |
| *CHRNG* | Multiple pterygium syndrome, lethal type | + | + | + | + | + | Full: Definitive |
| *CHST14* | Ehlers-Danlos syndrome, musculocontractural type 1 | - | - | + | - | + | Full: Definitive |
| *COG6* | Congenital disorder of glycosylation, type III | + | - | - | - | + | Full: Definitive |
| *CTSA* | Galactosialidosis | + | + | + | + | + | Secondary |
| *DHCR24* | Desmosterolosis | + | + | - | - | + | Full: Definitive |
| *DHCR7* | Smith-Lemli-Opitz syndrome | + | - | + | + | + | Secondary |
| *DOK7* | Myasthenic syndrome, congenital, 10 | - | + | + | + | + | Secondary |
| *ENPP1* | Arterial calcification of infancy 1 | + | + | - | + | + | Secondary |
| *EPHB4* | Capillary malformation-arteriovenous malformation 2; Lymphatic malformation 7 | + | + | - | - | + | Full: Definitive |
| *ETFA* | Glutaric acidemia IIA | - | - | + | - | + | Secondary |
| *ETFB* | Glutaric acidemia IIB | - | - | + | - | + | Secondary |
| *ETFDH* | Glutaric acidemia IIc | - | - | + | - | + | Secondary |
| *FAM111A* | Kenny Caffey; Gracile bone dysplasia | - | - | + | - | + | Full: Definitive |
| *FAM20C* | Raine syndrome | - | - | + | + | + | Full: Definitive |
| *FBXL4* | Mitochondrial DNA depletion syndrome 13 | - | - | + | - | + | Secondary |
| *FIG4* | Charcot-Marie-Tooth disease, type 4J; Yunis-Varon syndrome; Polymicrogyria | + | & | - | - | & | Secondary |
| *FKTN* | Myopathy, muscular dystrophy dystroglycanopathy | - | - | + | - | + | Secondary |
| *FLNB* | Atelosteogenesis, type 1 and 3; Boomerang dysplasia; Larsen syndrome | - | + | + | + | + | Full: Definitive |
| *FLT4* | Lymphatic malformation 1 | + | + | - | - | + | Full: Definitive |
| *FLT4* | Congenital heart defects, multiple types, 7 | - |  | - |  | + | Full: Definitive |
| *FLVCR2* | Proliferative vasculopathy and hysranencphaly-hydrocephaly syndrome | - | - | + | + | + | Full: Definitive |
| *FOXC2* | Lymphedema-distichiasis syndrome | + | + | - | - | + | Full: Definitive |
| *FOXP3* | Immunodysregulation, polyendocrinopathy, and enteropathy, X-linked | + | + | + | - | + | Full: Definitive |
| *GALNS* | Mucopolysaccharidosis IVA | + | + | - | - | + | Secondary |
| *GBA1* | Gaucher disease, type I | + | + | + | + | + | Secondary |
| *GBE1* | Glycogen storage disease IV | + | + | + | - | + | Full: Definitive |
| *GLDN* | Lethal congenital contracture syndrome 11 | - | + | + | + | + | Full: Definitive |
| *GNPTAB* | Mucolipidosis | + | - | - | - | + | Secondary |
| *GRIP1* | Fraser syndrome 3 | + | + | + | + | + | Full: Definitive |
| *GUSB* | Mucopolysaccharidosis VII | + | + | + | - | + | Secondary |
| *HYLS1* | Hydrolethalus syndrome | - | - | + | + | + | Full: Moderate |
| *INVS* | Nephronophthisis 2, infantile | - | + | + | - | + | Secondary |
| *KIDINS220* | Ventriculomegaly and arthrogryposis | - | - | + | + | + | Full: Definitive |
| *LIPA* | Lysosomal acid lipase deficiency | + | + | - | - | + | Secondary |
| *MGAT2* | Congenital disorder of glycosylation, type IIa | + | - | - | - | & | Secondary |
| *MVK* | Mevalonate kinase deficiency | + | - | - | + | + | Secondary |
| *NEU1* | Sialidosis, type I and II | + | + | - | - | + | Secondary |
| *NIPBL* | Cornelia de Lange syndrome | - | - | + | - | + | Secondary |
| *NPC1* | Niemann-Pick disease, type C1 and D | + | + | - | - | + | Secondary |
| *PHGDH* | Neu-Laxova syndrome 1; Phosphoglycerate dehydrogenase deficiency | + | - | + | + | + | Secondary |
| *PIEZO1* | Lymphatic malformation 6 | + | + | + | - | + | Full: Definitive |
| *PIEZO1* | Dehydrated hereditary stomatocytosis with or without pseudohyperkalemia and/or perinatal edema | + | + | - | - | + | Full: Moderate |
| *PKLR* | Pyruvate kinase deficiency | + | + | - | + | + | Full: Definitive |
| *PMM2* | Congenital disorder of glycosylation, type Ia | + | + | - | - | + | Secondary |
| *RAF1* | Noonan syndrome 5 | + | + | + | + | + | Secondary |
| *RNU4ATAC* | Lowry-Wood syndrome; Microcephalic osteodysplastic primordial dwarfism, type I; Roifman syndrome | - | - | + | - | + | Secondary |
| *SEC23B* | Dyserythropoietic anemia, congenital, type II | + | + | + | + | + | Full: Definitive |
| *SGPL1* | Nephrotic syndrome 14 | + | + | + | - | + | Full: Definitive |
| *SLC17A5* | Sialic acid storage disorder, infantile | + | + | + | - | + | Secondary |
| *SLC35D1* | Schneckenbecken dysplasia | - | - | + | + | + | Full: Definitive |
| *SMPD1* | Niemann-Pick disease, type A; Niemann-Pick disease, type B | + | & | - | - | - | Secondary |
| *SUMF1* | Mucosulfatidosis | + | + | - | - | - | Secondary |
| *TAZ* | Barth syndrome | + | (+) | + | (+) | + | Secondary |
| *TRIP11* | Achondrogenesis, type IA; Odontochondrodysplasia 1 | + | - | + | + | + | Full: Definitive |
| *TUBGCP6* | Microcephaly and chorioretinopathy, autosomal recessive | - | - | - | - | + | Full: Definitive |
| *WDFY3* | Microcephaly 18, primary, autosomal dominant | + | - | - | - | - | Secondary |
