# Supplementary material for: Curating the Fetal Genome: Experience of the ClinGen Prenatal Gene Curation Expert Panel (GCEP)
Source: Prenat Diagn. 2026 Sep 23;46(11):1703–10. doi: 10.1002/pd.70246 (PMC13659245; doi:10.1002/pd.70246)
Supplement: Supplementary file 2 [file PD-46-1703-s002.docx]

**Prenatal GCEP ClinGen Publication Acknowledgements**

Participating members:

| **First Name** | **Last Name** | **Role** | **Institution** |
| --- | --- | --- | --- |
| Shagun | Aggarwal | Biocurator | Nizam's Institute of Medical Sciences |
| Caitlin | Baptiste | Expert | Columbia University Medical Center |
| Brenna | Boyd | Biocurator | Columbia University Medical Center |
| Amanda | Buchanan | Biocurator | Tufts Medical Center |
| Alicia | Byrne | Expert | Broad Institute of MIT and Harvard |
| Imen | Chakchouk | Biocurator | Baylor College of Medicine |
| David | Chitayat | Expert | Mt Sinai Toronto |
| Lyn | Chitty | Expert | Great Ormond Street Hospital for Children NHS Foundation Trust |
| Pinal | Dalwadi | Coordinator | Geisinger |
| Avinash | Dharmadhikari | Biocurator | Children's Hospital Los Angeles |
| Lilian | Downie | Biocurator | Murdoch Children’s Research Institute |
| Stephanie | Dukhovny | Expert | Oregon Health and Sciences University |
| Mythily | Ganapathi | Biocurator | Columbia University Medical Center |
| Kelly | Gilmore | Biocurator | University of North Carolina |
| Elena | Greenfeld | Expert | Mount Sinai Health System |
| Lylach | Haizler-Cohen | Expert | Children’s Mercy Hospital |
| Vaidehi | Jobanputra | Biocurator | New York Genome Center |
| Gabrielle | Litteken | Coordinator | Geisinger |
| Hannah | Perrin | Biocurator | University of California San Francisco |
| Julie | Ratliff | Biocurator | Broad Institute of MIT and Harvard |
| Rebecca | Reimers | Expert | Rady Children’s Institute for Genomic Medicine |
| Erin | Riggs | Coordinator | Geisinger |
| Teresa | Sparks | Expert | University of California San Francisco |
| Samantha | Stover | Biocurator | Vanderbilt University Medical Center |
| Amanda | Thomas-Wilson | Biocurator | New York Genome Center |
| Leandra | Tolusso | Biocurator | Cincinnati Children’s Hospital Medical Center |
| Neeta | Vora | Expert | University of North Carolina |
| Tsegaselassie | Workalemahu | Biocurator | University of Utah |
| Roni | Zemet-Lazar | Biocurator, Expert | Baylor College of Medicine |
